# Annual dementia incidence and monetary burden attributable to fine particulate matter (PM_2.5_) exposure in Sweden

**DOI:** 10.1186/s12940-021-00750-x

**Published:** 2021-05-27

**Authors:** Hedi Katre Kriit, Bertil Forsberg, Daniel Oudin Åström, Anna Oudin

**Affiliations:** 1 Department of Public Health and Clinical Medicine, Section of Sustainable Health, Umeå University, Umeå, Sweden; 2 Division of Occupational and Environmental Medicine, Department of Laboratory Medicine, Lund University, Tornblad Institute, Biskopsgatan 7, 223v62, Lund, Sweden

**Keywords:** Dementia, Incidence, Particulate matter, Air pollution, PM_2.5_, Societal costs, QALY

## Abstract

**Background:**

Alzheimer’s disease (AD) and other dementias currently represent the fifth most common cause of death in the world, according to the World Health Organization, with a projected future increase as the proportion of the elderly in the population is growing. Air pollution has emerged as a plausible risk factor for AD, but studies estimating dementia cases attributable to exposure to fine particulate matter (PM_2.5_) air pollution and resulting monetary estimates are lacking.

**Methods:**

We used data on average population-weighted exposure to ambient PM_2.5_ for the entire population of Sweden above 30 years of age. To estimate the annual number of dementia cases attributable to air pollution in the Swedish population above 60 years of age, we used the latest concentration response functions (CRF) between PM_2.5_ exposure and dementia incidence, based on ten longitudinal cohort studies, for the population above 60 years of age. To estimate the monetary burden of attributable cases, we calculated total costs related to dementia, including direct and indirect lifetime costs and intangible costs by including quality-adjusted life years (QALYs) lost. Two different monetary valuations of QALYs in Sweden were used to estimate the monetary value of reduced quality-of-life from two different payer perspectives.

**Results:**

The annual number of dementia cases attributable to PM_2.5_ exposure was estimated to be 820, which represents 5% of the annual dementia cases in Sweden. Direct and indirect lifetime average cost per dementia case was estimated to correspond € 213,000. A reduction of PM_2.5_ by 1 μg/m^3^ was estimated to yield 101 fewer cases of dementia incidences annually, resulting in an estimated monetary benefit ranging up to 0.01% of the Swedish GDP in 2019.

**Conclusion:**

This study estimated that 5% of annual dementia cases could be attributed to PM_2.5_ exposure, and that the resulting monetary burden is substantial. These findings suggest the need to consider airborne toxic pollutants associated with dementia incidence in public health policy decisions.

## Introduction

In urban areas particularly, air pollution is a major cause of morbidity and mortality across the world. At the same time, the number of people worldwide living in urban areas is projected to increase from 55% today to 68% by 2050 according to the United Nations’ (UN) Department of Economic and Social Affairs [1]. Specifically, for Sweden, 85% of the population is currently living in urban areas, with an expected increase in the future [2]. For example, ambient (outdoor) air pollution is estimated to kill 4.2 million (M) people worldwide, and 7600 people in Sweden every year [3, 4]. World Health Organization (WHO) data furthermore shows that 9 out of 10 people worldwide breathe air that exceeds WHO guidelines for an upper limit to external air pollutants [5]. Exposure to air pollution increases the risk for cardiovascular and respiratory diseases and for cancer [6]. During recent years, results from a growing number of studies indicate that air pollution influences not only cardio-respiratory health and cancer but may also increase risk for cognitive decline and risk for development of Alzheimer’s disease (AD) and other dementias [7]. AD and other dementias currently represent the fifth most common cause of death in the world according to WHO [8] with a projected future increase as the proportion of the elderly in the population is growing [9]. It is thus imperative to identify modifying factors. The Lancet Commission of 2020 added air pollution as a modifiable risk factor to the 2017 Lancet Commission on dementia prevention, intervention, and care life-course model, which comprised nine factors (lower education, hypertension, hearing impairment, smoking, obesity, depression, physical inactivity, diabetes, and infrequent social contact) [10].

The plausible pathways to support an association with exposure to particles include their potential to induce inflammatory responses, microglial activation, and production of reactive oxygen species, but also their potential to reach the brain directly via the olfactory bulb. The presence of early pathology in the olfactory bulb of AD patients and early olfactory dysfunction, which may precede brain alterations, suggest a potential role of inhaled toxins as a risk factor for AD [11]. Particulate matter (PM) that originates from combustion comprises fine or ultrafine particles (< 2.5 μm), shown as PM_2.5,_ tends to have a higher oxidative capacity and causes more damage than coarser particles that originate from other sources such as windblown soil and road dust [12].

Results from several studies have suggested a positive association between PM and AD and other dementias. Already in 2008, Calderón-Garcidueñas et al. suggested that long-term air pollution exposure was associated with neuroinflammation, an altered innate immune response, disruption of the blood-brain barrier, ultrafine particulate deposition, and accumulation of amyloid beta-42 and alpha-synuclein [13]. Jung et al. 2015 later conducted a prospective cohort study, where exposure assessment was done at postcode level based on 70 measuring stations across Taiwan [14]. They found that for an increase of 4.34 μg/m^3^ in PM_2.5_ exposure, increased their risk of being diagnosed with AD by 138% [14]. In the same year, Wu et al. 2015 carried out a case control study (249 cases and 497 controls) in the same population, finding an association between long-term exposure to high concentrations of particulate matter < 10 μm (PM_10_) and AD based on 24 urban measuring stations in the Taipei-Keelung metropolitan area [15]. In Northern Sweden, our group have found associations between AD as well as vascular dementia and individual-level exposure to local sources of traffic-related air pollution and wood-burning in a longitudinal cohort studies [16, 17]. Carey et al. 2018 conducted a retrospective cohort study in England including a total of 130,978 people aged between 50 and 79 years [18]. They observed that exposure to concentrations of PM_2.5_ greater than 16.3 μg/m^3^ (the highest quintile) was related to an increased risk of AD with a hazard ratio (HR) of 1.42 compared to the lowest quintile [18]. A large cohort study conducted in Canada (with a population of more than two million people, aged between 55 and 85 years), found that exposure to PM_2.5_ was associated with an increased risk of dementia, with a HR of 1.06 for an increase of 4.8 μg/m^3^ of PM_2.5_ as urban background (1 km^2^ squares as spatial resolution) [19]. In a review from 2019 the authors conclude that while the evidence from longitudinal cohort studies points toward an association between exposure to PM_2.5_ and increased risk of dementia, the exposure measure varies between studies, from local levels, to the urban background to the regional background [20]. Later, Yu et al. 2020 performed a meta-analysis, and estimated a pooled relative risk for dementia of 1.08 for a 5 μg/m^3^ increase in PM_2.5_ [7].

In Europe, the annual dementia-related societal costs including all costs occurring due to dementia, regardless of the funding source, have been estimated to be €10.3 billion (B), where informal care contributes about one third of the total costs. In Sweden, dementia societal annual costs were estimated to be approximately €6.3 M and most of these costs occur in social care sector [21]. The monetary burden of AD in Europe is also expected to increase with the growing number of people at risk giving an increased incidence rate. By 2050, the monetary burden is estimated to be almost twice the size compared with 2010 [22]. Given the heavy toll of dementia on individuals and society, as well as the ubiquitous nature of air pollution, the monetary burden of air pollution induced dementia should be recognised. If air pollution indeed increases the risk for dementia, then total costs due to air pollution attributed disease burden have been largely underestimated, as have the recognised causes of dementia.

The aim of the current study was to estimate the number of annual dementia cases attributed to PM_2.5_ exposure and the resulting monetary burden, in Sweden.

## Methods

### Population exposure to fine particulate matter (PM_2.5_)

Population exposure to nitrogen dioxide (NO_2_) and PM_2.5_ in ambient air for the years 2005, 2010 and 2015 has been estimated in a Swedish national model and expressed as population weighted means for all ages and specific age groups from 1 km grids [4]. These means were calculated by linking gridded population data with concentration layers. We apply the estimated mean population PM_2.5_ exposure obtained using the distribution of residents living in Sweden above 30 years of age. This number should be representative for the group 60+ (which are under risk to be dementia cases according to our definition) as the differences in exposure between age groups were small according to the air pollution model we used [4]. According to the national assessment, this weighted concentration of PM_2.5_ was 8.3 μg/m^3^ in 2015 (See Table 1.).

**Table 1:** Input data for the model

Parameter	Value	Description	Reference
**Health data**			
Population at risk	2,650,000	≥60 years old in Sweden 2019	Statistics Sweden [23]
Baseline incidence	5.77 (2.70–8.69)	Crude incidence rate per 1000 person-years	Van Bussel et al. 2017 [24]
Average duration of dementia	6 years		Vermunt et al. 2019 [25]
Concentration Response Function (CRF) for total PM_2.5_	1.08 (1.03–1.13) per 5 μg/m^3^	Meta-estimate	Yu et al. 2020 [7]
**Exposure data**			
Total PM_2.5_ concentration (C)	8.3 μg/m^3^		Gustafsson et al. 2018 [4]
**Economic data**			
Sum of direct and indirect costs ^a^	€ 40,000	Annual average costs per dementia case	Wimo et al. 2017 [21]
Medical care ^b^	€ 2000		
Social care sector ^c^	€ 31,000		
Indirect costs	€ 100		
Informal care	€ 6000		
Utility weights among dementia patients	0.457–0.486	EQ-5D	Mesterton et al. 2010 [26]
Utility weights for general population	0.795–0.845	EQ-5D	Burström et al. 2001 [27]
QALY-1 valuation	€ 226,600	Willingness-to-pay	Olofsson & Persson, 2018 [28]
QALY-2 valuation	€ 47,200	Threshold	Socialstyrelsen, 2011 [29]

^a^ Direct and indirect costs – sum of annual medical care, social care costs, indirect costs, informal costs

^b^ Funding body: county councils

^c^ Funding body: municipalities

### Concentration response function (CRF)

In the current study we used the CRF for PM_2.5_ exposure and cognitive impairment (mainly consisting of Alzheimer’s disease and other dementias), reported by Yu et al. 2020 (See Table 1.), who calculated a meta-estimate from ten longitudinal cohort studies [7]. No statistically significant associations between nitrogen dioxide/nitrogen oxides (NO_2_/NO_x_) or ozone (O_3_) and cognitive impairment were found. In the present study we estimate incident dementia cases and total costs attributed solely to PM_2.5_, partly because there is still substantial uncertainty regarding the independent effects of NO_2_/NO_x_ and O_3_, and partly to avoid the risk of double counting. It should be noted that the heterogeneity between the pooled studies in Yu et al. 2020 [7] was substantial. The results should therefore be interpreted with caution but are in line with what has been observed in several large high-quality longitudinal observational studies [18, 19]. It should also be mentioned that Yu et al. 2020 [7] calculated a meta-estimate from European studies that showed a considerably higher risk increase, but it became unprecise (not statistically significant). We chose to use the pooled estimate from all regions to conduct this study which corresponded to 1.08 per 5 μg/m^3^ increase in PM_2.5_.

### Baseline data

A Swedish cohort (Betula) has been used previously by our team to estimate the association between exposure to air pollution and dementia [17]. As the recruitment to the Betula study took place with a narrow-age-cohort design, the age distribution in the Betula study is not representative of the age-distribution in this study population. Moreover, the Betula study was relatively limited in size, and the dementia diagnostic procedure was not representative of the diagnostic procedure applied for the general population. We therefore used the baseline incidence from Bussel et al. (2017) instead [24], where data from a Dutch general practitioner registration network was used, which is representative of the general Dutch population. Dementia incidence cases was defined as International Classification of Primary Care 2nd Edition (ICPC-2) Code P70 (senile dementia/Alzheimer disease) among people 60 years or older during 1992–2014. Incidence rate was calculated for the following age groups: 60–64, 65–69, 70–74, 75–79, 80–84 and ≥ 85 years of age. In total 23,186 cases of dementia and 4,020,550 person-years were observed. Age group stratified crude mean incidence rate ratios were reported and were used as baseline incidence rate in this study.

### Dementia incidence cases attributable to PM_2.5_ calculation

The dementia incident cases (Inc) attributable to PM_2.5_ exposure was calculated as follows (Eq. 1.):

1 $$ Inc={Inc}_0\ast \left(1-{e}^{-\beta \ast x}\right) $$

Here, *Inc* is the incidence of interest, Inc_0_ is the baseline incidence for the outcome (here, age-dependent), β is the coefficient of the CRF, here log (RR), and x is the population weighted concentration of the exposure measure (here, modelled levels of PM_2.5_). We applied the CRF to individuals ≥60 years of age.

### Direct and indirect cost estimation

To be able to estimate the direct and indirect costs of dementia a literature search using the PubMed search engine was conducted using the following search string: “cost of disease” OR “societal cost” OR “cost of illness” AND (“dementia” OR “Alzheimer”) AND “Sweden”. The following inclusion criteria were applied: a) published between 2005 and 2020; b) using register data and conducted in Sweden; c) outcome defined by ICD-code; d) costs representing the health outcome in question; e) reporting both direct and indirect costs. If more than one study fulfilled the listed criteria the newest and most sufficient study was selected.

As a result of the literature search a study by Wimo et al. (2017) was chosen [21]. (See Table 1.) Wimo et al. (2017) conducted a prevalence-based cost-of-illness (COI) study from a societal perspective to estimate the average annual cost burden of dementia in Sweden. Wimo et al. (2017) report detailed cost estimates for both annual direct and indirect costs. Direct costs included average costs estimates per dementia case that occur in the medical care sector (e.g. hospital visits, emergency care, primary care physician etc.), which are financed by the county councils; social care sector (e.g. institutional care, day care, home services etc), which are financed by municipalities and estimates of informal care (e.g. caregiver time). Indirect costs reflected the average productivity losses based on average wage among 55–65 years old. In this paper we refer to annual direct costs as a sum of medical care and social care and indirect costs as a sum of informal care and indirect cost per dementia case, as reported by Wimo et al. (2017) and used to illustrate the average annual direct and indirect costs in respective categories due to dementia incidences attributable to PM_2.5_. Intangible costs were not included in that study.

### Calculating the direct and indirect lifetime costs of a dementia case

Since dementia is a chronic disease, this suggests that the direct and indirect costs occur yearly throughout the disease duration. For the dementia disease duration, we used an estimate of 6 years, retrieved from a study conducted by Vermunt et al. 2019 [25]. They used a longitudinal prospective multinational and -cohort study to estimate the duration of dementia. To estimate the average direct and indirect lifetime cost of each dementia case, the annual direct and indirect costs were discounted using the rate of 3.5% based on formula applied by the Swedish Transport Agency [30] and then summed over the disease duration years (Eq. 2.). All direct and indirect costs were inflated to 2019 values and reported in €_2019_ values (1 € = 10,5891 SEK) [31], any slight differences in cost estimates are due to rounding.

2 $$ F=\sum \frac{D+I}{\kern0.5em {\left(1+r\right)}^n} $$

*F = sum of direct and indirect lifetime costs per dementia case.*

*D = sum of annual average direct costs per dementia case.*

*I = sum of annual average indirect costs per dementia case.*

*r = discount rate.*

*n = average disease duration in years.*

### Intangible costs associated with loss of quality of life

A quality-adjusted life-year (QALY) measure was used to account for the intangible costs associated with loss of health-related quality of life due to dementia, which is often a neglected category of costs in health economic assessments. By including the effects of dementia on health-related quality of life, we were able to measure and monetize the life-quality losses due to dementia compared to the general population in the same age groups.

### Valuation of quality-of-life losses

Two different monetary estimates were used to monetize the health-related quality of life losses to illustrate the different budgets constraints faced in decision-making processes among the potential funders of PM_2.5_ amelioration.

Firstly, the Swedish Transport Agency takes into account the health burden associated with traffic induced PM_2.5_ exposure as external costs in their cost-benefit analysis (CBAs), as a component when estimating the net monetary costs of planned infrastructure. The Swedish Transport Agency assesses the costs and benefits from the societal perspective, where the state is the funding body. The valuation of health burden is typically based on questionnaire studies estimating the willingness-to-pay (WTP) for reduction in risk for fatality or non-fatal injuries in traffic [30]. At the present time, the WTP for a risk reduction of non-fatal injury applied by the Swedish Transport Agency, is based on a Swedish population-based web-based questionnaire study, that applies a chain approach to estimate the monetary value of a QALY [32]. This was reported to be € 226,600 [28]. This is referred to as the QALY-1 valuation in this study.

Secondly, we use the threshold value of a QALY €47,200 commonly applied in cost effectiveness analyses (CEAs) in the Swedish health care sector (e.g., The Dental and Pharmaceutical Benefits Agency) to determine the cost effectiveness of a drug or an intervention. Therefore, we use this threshold value, which is often stated as ‘rule-of-thumb’ in the Swedish policy context [29], to illustrate the monetary value applied in decision-making where the healthcare is the funding body. This is referred to as the QALY-2 valuation in this study.

In the Swedish context, Persson and Olofsson have argued [28], that the health sector and The Dental and Pharmaceutical Benefits Agency should raise the threshold value up to the value of € 226,600, which is based on the latest research estimating the individual WTP for a QALY. This is in contrast to the current threshold value for a QALY applied in the health care sector, whose origins is unclear, but has nevertheless maintained its usage over the past decades. Due to this current debate, we include both QALY valuations applied in the Swedish policy-context to facilitate the knowledge transfer from our interdisciplinary research to decision-making tools currently used in the respective sectors.

### Monetizing the quality-of-life losses

The lost QALYs were calculated as the differences between the age-specific utility weights among the general population and dementia patients (Eq. 3). This difference was multiplied by the duration of dementia (See Table 1.) to obtain the average number of lost QALYs among dementia patients. To monetize the quality-of-life losses the average number of lost QALYs where then multiplied by the current value applied in the transport sector (1) and in the health care sector (2). Discounting was not applied to estimate the monetary value of QALY losses.

3 $$ {Q}_{1,2}=\left(\left({U}_1-{U}_2\right)\ast t\right)\ast {V}_{1,2}\Big) $$

*Where:*

Q_1,2_ = monetized value of quality-of-life losses.

U_1_ = utility weights among general population.

U_2_ = utility weights among dementia patients.

t = average duration of dementia.

V_1,2_ = monetary valuation of quality-of-life losses based on 1) QALY-1 valuation 2) QALY-2 valuation.

### Total cost (TC) of dementia estimates

The total cost (TC_1,2_) of dementia is estimated as the sum of direct and indirect lifetime costs (F) and monetary valuation of average loss of QALY’s (Q_1,2_) among dementia patients (Eq. 4). We use this total cost to reflect the monetary burden attributable to PM_2.5_ exposure.

4 $$ {TC}_{1,2}=F+{Q}_{1,2} $$

### Hypothetical scenario

In addition, we investigated what the potential impact of reducing the levels of PM_2.5_ by 1 μg/m^3^ would be on the number of incident dementia cases and the respective direct and indirect lifetime costs and total costs of dementia.

### Two-sided sensitivity analysis

In order to illustrate the uncertainty, when it comes to CRF and direct and indirect lifetime cost estimates, we conducted a two-sided sensitivity analysis. The confidence interval reported by Yu et al. 2020 [7], was used for to illustrate the uncertainty of the annual cases of dementia incidence attributable to PM_2.5_ exposure. Since, no confidence intervals were available for the cost estimates, we assumed a cost variation of ±20%, as suggested as an alternative method by Briggs et al. 2012 [33]. The resulting uncertainty was reported after the mean estimates, in parenthesis. The monetary values of a QALY were however treated as fixed.

### Unmeasured confounding

We calculated the E-value (Eq. 5) to evaluate the impact which an unmeasured confounder would need to have on both exposure and outcome for the observed meta-estimate relative risk to be 1 [34]. Since the meta-estimate from Yu et al. 2020 [7] were based on only ten studies, we calculated the E-value for the meta-estimate as well as for the lower bound of the 95% confidence interval [35].

5 $$ Evalue= HR+\surd \left( HR\times \left( HR-1\right)\right) $$

## Results

According to our definition of the study population, including people 60 years or older, there were 2.6 million (M) people at risk for dementia in Sweden 2019 (See Table 1.). We estimated the number of annual dementia cases attributable to PM_2.5_ exposure to be 820 (See Table 2.), which represents 5% of the annual dementia incidence in Sweden. Annual direct and indirect lifetime costs are estimated to correspond to €32,9 M, out of which 78% are estimated to occur in the social care sector.

**Table 2:** Annual dementia cases attributable to total PM_2.5_ and average annual direct and indirect lifetime costs (millions/€_2019_)

	Dementia cases attributable to PM_2.5_ and average direct and indirect annual costs in M/€_2019_	% of total number of cases and % of given costs within direct and indirect costs
Attributable dementia cases in Sweden	820 (320 – 1282)	5% (2–8%)
Direct and indirect lifetime costs ^a^	€32,9 (€10,3 – 61,6)	100%
Medical care costs	€1,5 (€0,5 – 2,8)	5%
Social care sector costs	€25,7 (€8 – 48,2)	78%
Indirect costs	€0,1 (€0,02 – 0,1)	0,2%
Informal care	€5 (€1,5 – 9,3)	15%

^a^ Direct and indirect lifetime – sum of medical care, social care costs, indirect costs, informal costs

Direct and indirect average lifetime costs per dementia case is estimated to correspond to €213,000 (170,600-256,000) (See Table 3.).

**Table 3:** Total costs of dementia cases attributable to PM_2.5_ using direct and indirect lifetime costs of dementia and two different valuations to monetize quality-of-life losses among dementia patients

	Direct and indirect lifetime costs per dementia case	Total costs – 1 (TC_1_)^a^	Total costs – 2 (TC_2_)^b^
Cost per case of dementia	€213,000 (€171,000 - 256,000)	€690,000 (€645,000 - 730,000)	€312,000 (€269,000 - 355,000)
Monetary burden (M/€_2019_) of dementia cases attributable to PM_2.5_	€189 (€94 – 283)	€567 (€283 – 850)	€274 (€123 – 406)

^a^ TC_1_Total costs - sum of direct and indirect lifetime costs (discount rate 3.5%) and monetary valuation of QALY’s lost among dementia patients using QALY-1 value, representing societal perspective

^b^ TC_2_ Total costs - sum of direct and indirect lifetime costs (discount rate 3.5%) and monetary valuation of QALY’s lost among dementia patients using QALY-2 value, representing health care perspective

On average, a person with dementia is estimated to lose 2.091 QALY’s compared to the general population in the same age, given the average duration of disease. Total costs per dementia case were estimated to correspond to €619,000, when monetizing the QALY losses with QALY-1 and €312,000 with QALY-2 value, respectively (See Table 3.).

The direct and indirect lifetime costs of the dementia burden attributable to PM_2.5_ exposure were estimated to correspond on average to €189 M. A sum of 1715 (670–2680) QALYs were estimated to be lost on average among the annual dementia incidences attributable to PM_2.5_ exposure. Total costs of the dementia burden attributable to PM_2.5_ were estimated to correspond on average to €567 M and €275 M when using a monetary value of QALY-1 and QALY-2, respectively. The direct and indirect lifetime costs as a proportion of total costs approximated 34% and 69% for TC_1_ and TC_2_, respectively. The average annual individual value among the population at risk was estimated to correspond to €72, €215 and €104 for direct and indirect lifetime costs, TC_1_ and TC_2_, respectively.

We then calculated the effect on dementia incidence and costs by decreasing exposure by 1 μg/m^3^ and estimated a 12% decrease in annual cases of dementia attributable to PM_2.5_ exposure, approximately 1% of total number of incident cases (See Table 4.). The annual reduction in direct and indirect lifetime costs was estimated to be 12%, corresponding to €4 M, where the largest effect of reduction of €3 M was estimated to occur in social care. The reduction in dementia incidences was estimated to correspond to a reduced monetized burden of €19 M, €66 M and €28 M for direct and indirect lifetime costs, TC_1_ and TC_2_, respectively (See Table 5). The average annual individual value among the population at risk was estimated to correspond to €7, €25 and €11, for direct and indirect lifetime costs, TC_1_and TC_2_, respectively.

**Table 4:** Estimated reduction in annual number of dementia cases and direct and indirect lifetime costs (millions/€_2019_) if 1 μg/m^3^ reduction in PM_2.5_ annual exposure

	Estimated reduction in cases of dementia incidence and averted monetary values (M/€)	% from annual case incidence and estimated costs compared to main analysis
Attributable incident cases in Sweden	101 (39–160)	1% (0,3% – 1,1%)
Direct and indirect lifetime costs ^a^	€4 (€1,2 – 7,7)	12%
Medical care costs	€0,2 (€0,1 – 0,38)	12%
Social care sector costs	€3 (€0,9 – 6)	12%
Indirect costs	€0,008 (€0,002 – 0,01)	12%
Informal care	€0,66 (€0,2 – 1,13)	12%

^a^ Direct and indirect lifetime costs – sum of medical care, social care costs, indirect costs, informal costs

**Table 5:** Estimated reduction of monetized dementia burden in (millions/€_2019_) if assuming 1 μg/m^3^ reduction in PM_2.5_

	Estimated monetary values of the reduced dementia burden (M/€)	% of annual monetary values of reduced dementia burden
Direct and indirect lifetime costs	€19 (€9 – 38)	15%
Total costs – 1 (TC_1_) ^a^	€66 (€28 – 113)	13%
Total costs – 2 (TC_2_) ^b^	€28 (€13 – 57)	14%

^a^ TC_1_ Total costs - sum of direct and indirect lifetime costs (discount rate 3.5%) and monetary valuation of QALY’s lost among dementia patients using QALY-1 value, representing societal perspective

^b^ TC_2_ Total costs - sum of direct and indirect lifetime costs and monetary valuation (discount rate 3.5%) of QALY’s lost among dementia patients using QALY-2 value, representing health care perspective

The annual direct and indirect lifetime costs attributable to PM_2.5_ dementia burden could vary sixfold (See Table 2.). The direct and indirect lifetime cost per dementia case was estimated to vary between €171,000 and €256,000. The direct and indirect lifetime monetary burden lower bound was estimated to be approximately 50% lower and upper bound to be 60% higher compared with the mean value of €189 M, respectively (See Table 3). The total cost of estimated dementia monetary burden variation lower bound was approximately 49% lower and the upper bound was estimated to be 67% higher compared to the average estimate applying QALY-1 valuation (See Table 3.).

We did not find strong evidence for unmeasured confounding. The E-values for the observed meta-estimate and the lower bound of the 95% CI were 1.37 and 1.21 respectively.

## Discussion

To the best of our knowledge, this is the first health economic assessment reporting the annual dementia incidence and monetary burden attributable to PM_2.5_ exposure. In this study, we estimated the annual dementia incidence attributable to PM_2.5_ exposure to be 820 in Sweden, which corresponds to 5% of the yearly dementia incidence. The total costs for the dementia disease burden attributed to PM_2.5_ were estimated to correspond to €215 and €104 per individual at risk, based on QALY-1 and QALY-2 valuation, respectively.

A reduction of 1 μg/m^3^ of PM_2.5_ exposure was estimated to reduce annual dementia incidence by 101 cases, which corresponds to an estimated monetary reduction of €19 M in direct and indirect lifetime costs. This corresponds to a decrease of 13–15% of the annual monetary dementia burden attributable to PM_2.5_ exposure compared to the main analysis.

Pimpin et al. 2018 estimated the cumulative dementia incidence in 2017 due to NO_2_ exposure, which was estimated to be 5008 cases in England, corresponding to €30 M as health care costs, where €28 M was estimated to occur in social care sector [36]. Pimpin et al. 2018 derived their medical and social cost estimates for dementia from register data and previously published literature. These results are not directly comparable to our estimates, since we used CRF for PM_2.5_ and an age group-specific incidence rate ratio to estimate the cases attributable to PM_2.5_ exposure, whereas Pimpin et al. 2018 used prevalence-based and mortality data to estimate the attributable fraction of dementia disease to NO_2_. Even though a statistically significant association between NO_2_ exposure and dementia was found in some studies [16, 18, 19, 37], a meta-analysis found no significant association [7]. We chose to estimate dementia cases attributable solely to PM_2.5_ in the present study. Furthermore, in the study by Pimpin et al. 2018 [36], the UK National Health Service (NHS) economic impact reflects only costs including primary care, secondary care, social care, and medication related to dementia. Whereas, they ignore informal costs, indirect costs and intangible costs related to dementia disease, which contributes to underestimation of monetary burden of dementia compared to our study and partly explains the difference in between monetary burden estimates between studies.

In the present study, we used air pollution data where the concentration patterns of PM_2.5_ over Sweden were calculated with a 1 × 1 km grid resolution. According to the model, the major fraction of total PM_2.5_ consists of long range transported particles and only a small fraction consists of locally emitted particles; the long distance transported particles fraction of PM_2.5_ was 7.2 μg/m^3^ in average whereas the domestic heating from wood fuel contributed to on average of 0.8 μg/m^3^ to the annual mean of PM_2.5_ in 2015 [4]. Reducing PM_2.5_ by 1 μg/m^3^, which according to our estimations of direct and indirect lifetime costs correspond to 0.004% of Swedish gross domestic product (GDP) due to reduced burden of dementia. Thus, it would require a meaningful intervention on a local level, since 1 μg/m^3^ is a substantial proportion of the local contribution to PM_2.5_. It should be noted however that, if our assumptions are correct, even smaller decreases than 1 μg/m^3^ would have substantial health and economic effects. It is furthermore possible that the effect size following a specific reduction in mass concentration may depend on the type of PM that is removed.

There is increasing evidence that locally emitted air pollutants have substantially larger health effects compared to regional air pollution. Recently, a few meta-analyses have focused specifically on analysing the importance of local pollution sources such as traffic and differences in concentrations within cities and have described the relationships at relatively low concentrations [38–40]. In one of our previous studies, where we investigate the association between locally emitted particles and incidence of dementia, we observed a HR of 1.55 for a 1 μg/m^3^ increase in PM_2.5_ stemming from wood smoke [17]. This is notably much higher than the meta-estimate of the CRF estimated by Yu and colleagues of 1.08 [7]. The discrepancy is most likely because the studies included in that meta-analysis varied in terms of studying local, urban background or regional background PM_2.5_, which likely would have a substantial effect on the CRFs. In the present study however, we did not consider that CRFs may differ between local and regional PM_2.5_ in our estimations, as evidence for such differences are too sparse at present for PM_2.5_ and dementia. Future studies should further investigate differences in CRFs dependent on local or regional PM_2.5_.

Total direct and indirect lifetime costs of dementia burden attributable to PM_2.5_ were estimated to correspond to €33 M annually, with the social care sector having the highest cost burden (€25.7 M). In Sweden, medical care is financed by the regions and social care services by municipalities. The Swedish Association of Local Authorities and Regions (SALAR) reports that the social care sector (municipalities) allocated €12B for elderly care in 2019 [41], suggesting that 0.21% out of this budget was allocated to care for PM_2.5_ attributable dementia burden, based on this study estimates. Assuming a decrease of PM_2.5_ exposure by 1 μg/m^3^ the monetized dementia burden avoided would correspond to 0.025% of the social care budget in 2019. The regional budget responsible for medical care was €31B in 2019 [42]. Based on our study estimates, 0.005% of this was allocated to medical care expenses for dementia burden attributable to PM_2.5_. In total, direct and indirect lifetime costs attributable to dementia induced by PM_2.5_ exposure were estimated to correspond to 0.4% of the total healthcare budget (€7.5B) allocated by the Swedish Government in 2019 [43].

An important step towards complete health economic assessment of monetizing the dementia burden attributable to PM_2.5_ was made by including the QALY measure to account for the intangible costs, i.e. the value of the life quality losses due to dementia, which is a cost component that has been generally ignored by the COI studies in the past [44]. Nevertheless, some studies of health economic assessments valuing the intangible costs by including the QALY losses attributable to air pollution have recently been performed [45].

Use of the QALY measure facilitates easy knowledge transfer between interdisciplinary fields of research. For example, the QALY measure allows a comparison between planned interventions from both a health care perspective and societal perspective. In England, Schmitt (2015) [46] also used two different QALY values from two different funding perspectives (one for representing the National Health Service (NHS) budget constraints and the other representing consumption value of a QALY with the state as the funding body to monetize effects of air pollution attributable health effects. Similarly, to the Swedish context, the NHS QALY value is about five times lower (€13,500 [47] vs €67,000 [46] (inflated to €_2019_ values to ease the comparison), compared to consumption value of QALY, where tax rises are expected to fund the shortfall. In addition, Schmitt (2015) estimated the averted monetary benefits of reduced disease burden in a hypothetical scenario, where a reduction of PM_2.5_ concentrations by 1 μg/m^3^ was assumed [46]. Schmitt (2015) reports that an individual value per person at risk was €1368 and €248 from a private consumer and NHS perspective respectively, for reduced adverse health impacts due to a reduction by 1 μg/m3 in ambient PM_2.5_ in London [46]. These estimates are substantially higher and not directly comparable with the results from this study results, since they represent the monetary burden of three chronic health outcomes (i.e., cardiovascular disease, chronic obstructive pulmonary disease and lung cancer) over a 60-year time horizon.

In general, a survey-based willingness to pay estimate, which is often used in the environmental and transport sectors (as in the case of Swedish Transport Agency) is the preferred measure since it follows the welfare theory and consumer sovereignty. But as discussed by Olofsson et al. 2019, the survey-based value of a QALY is an important improvement, but not sufficient [48]. There has also been research, in Sweden analysing the Swedish Dental and Pharmaceutical Benefits Agency (TLV) historical reimbursements decisions on new drugs, where implications of higher willingness-to-pay thresholds for a QALY was found [49]. There is ongoing debate and research regarding different valuation methods of QALYs and the monetary values applied, which have shown to vary substantially [49, 50]. We wanted to illustrate the valuation of QALYs using values currently applied in decision-making process in Sweden, however we acknowledge that these values may vary between contexts and in time.

It is out of the scope of this study to discuss in depth the complex budget constraints faced by decision makers representing either health care or societal perspectives. This study presents total cost estimates that enable inclusion of the quantified monetary estimates of reduced air pollution in decision-making tools, using either CBAs or CEAs as the basis of decision-making in the respective field.

We acknowledge that this paper has important limitations that should be considered. Firstly, we used Wimo et al. (2017) COI study [21], that estimates the societal burden of dementia for one year based on dementia case prevalence, which might lead to underestimation of total disease burden. It has been recommended that incidence-based cost studies are used for improved policy support [44]. These studies measure the costs of illness from diagnosis until endpoint and thereby deliver a higher precision with respect to direct and indirect cost estimates [51]. Although ideal, there is a lack of incidence-base COI studies in general, and therefore, prevalence-based studies may be used to guide the health care budget planning [51].

Secondly, we lacked age-specific dementia baseline incidence estimates for the Swedish population and used estimates from a Dutch study instead [24], assuming that the incidence in the Swedish population would be similar. This might have led to some errors in the estimation, but data from the Swedish Quality Register on Dementia suggest that 150,000 people in Sweden currently have dementia and approximately 24,000 people develop the disease each year [52], which is very similar to our estimations. We therefore do not consider this assumption to cause any substantial errors in our estimates.

Thirdly, an underestimation of dementia monetary burden is possible since we use an average duration of moderate dementia to estimate the direct and indirect lifetime costs and QALY losses. But, depending on the age of onset and disease severity, the duration of disease might vary substantially [25] and, it is impossible to know exactly the detailed information on the onset age and severity of dementia attributable to PM_2.5_. Therefore, we consider that using an average duration to quantify the monetary burden of dementia to be reasonable.

Fourthly, we used individual WTP to monetize life quality losses (QALY-1), however this may not be the only source of information which decision makers rely upon. In addition, as noted previously, there is heterogeneity between the two valuation values used in the present study [53], which needs to be recognized if they are to be incorporated into decision-making tools.

Fifthly, it should be noted that since we chose to include dementia in people aged 60 years or older, this means that individuals in whom dementia occurs before age 60 are not included in the present study. It should furthermore be noted that the population-weighted average exposure of PM_2.5_ used in the present study was estimated for the population 30 years or older. Our assumption is thus that the exposure of persons 60 years or older is similar to the total population 30 years or older, which is supported by the report by Gustafsson et al. 2018, where it is shown that exposure differences between different age groups are small [4].

Sixthly, as the effect estimates used in the current study are derived from observational studies, we cannot rule out the potential impact of unmeasured confounding. However, based on the E-value calculation we believe that there is no strong evidence for the presence of an unmeasured confounder associatied with both exposure and outcome that could explain away the observed meta-estimate.

It should furthermore be noted that although the evidence for air pollution concentrations at the residential address to be linked with natural cause mortality and a range of health outcomes is mounting and very strong [54], such studies are always prone to exposure misclassification. For example, if exposure is estimated at the residential address, then work-place exposure, exposure during commuting or exposure due to indoor sources are not taken into account, which leads to exposure misclassification. When it comes to the newer research area on air pollution as a risk factor for dementia, evidence for a causal association is now strong enough according to the new Lancet commision [10], but the heterogeneity between effect estimates is high, most likely partly due to exposure measurement error and because the number of studies is low. It is likely that the CRF by Yu et al. 2020 [7] will be updated in due course, which is a natural process for a new research field and should not be seen as a cause for concern. The CRFs for air pollution and mortality have been updated several times, and are still being updated as the quality of air pollution studies is increasing and the number of studies as well. The CRF regarding total PM_2.5_ and dementia from the Swedish Betula-study has been published [55], but since that CRF was estimated for the local contribution of PM_2.5_ it should not be used to assess health impacts from total PM_2.5_.

## Conclusion

In conclusion, our results show that as much as 5% of annual dementia cases in Sweden could be attributed to PM_2.5_ exposure, where air pollution levels are quite low in an international perspective. PM_2.5_ attributable dementia monetary burden (TC-1) was estimated to correspond to 0.1% of Swedish GDP in 2019. The implications of these findings suggest the need to consider airborne toxic pollutants associated with dementia incidence in public health policy decisions.

## Data Availability

All data and material used for calculation is available in the manuscript.

## Abbrevations

AD: Alzheimer’s disease
B: Billion
CBA: Cost-benefit analysis
CEA: Cost-effectiveness analysis
CRF: Concentration-response function
COI: Cost of illness study
HR: Hazard ratio
GDP: Gross domestic product
QALY: Quality adjusted life-year
M: Million
NO_2_: Nitrogen dioxide
NO_x_: Nitrogen oxide
NHS: National Health Service
O_3_: Ozone
PM: Particulate matter
PM_2.5_: Fine particulate matter <2.5 μm
PM_10_: Particulate matter <10 μm
RR: Relative risk
SALAR: The Swedish Association of Local Authorities and Regions
TLV: Swedish Dental and Pharmaceutical Benefits Agency
UN: United Nations
WTP: Willingness to pay
WHO: World Health Organization
